# Molecular evolution of two asymptomatic echovirus 6 strains that constitute a novel branch of recently epidemic echovirus 6 in China

**DOI:** 10.1186/s12985-017-0809-2

**Published:** 2017-07-25

**Authors:** Hao Sun, Xiaoqin Huang, Keqin Lin, Kai Huang, Jiayou Chu, Zhaoqing Yang, Shaohui Ma

**Affiliations:** 1 The Department of Medical Genetics, Institute of Medical Biology, Chinese Academy of Medical Sciences and Peking Union Medical College (CAMS & PUMC), 935 Jiao Ling Road, Kunming, 650118 Yunnan Province People’s Republic of China; 2 Yunnan Key Laboratory of Vaccine Research Development on Severe Infectious Disease, Kunming, 650118 People’s Republic of China

**Keywords:** Enterovirus, Echovirus 6, Natural selection, Polygenetic tree, Recombination

## Abstract

**Background:**

Echovirus 6 (E6) infections are associated with aseptic meningitis and acute flaccid paralysis (AFP). But some infections, sometimes most of them, are asymptomatic. The mechanism of E6 virulence is unknown. Analyses of the molecular evolution of asymptomatic E6 may help understand why the infections show different manifestations.

**Methods:**

Ninety-six stool samples of healthy children in Yunnan, China were collected and two E6 strains were isolated from them. The whole genomes of these two E6 strains were sequenced, and their molecular evolution was analyzed.

**Results:**

The results showed that the two E6 strains may be derived from KJ7724XX strains, which were predominant in AFP patients in Shangdong in 2011. The evolution was accelerated when the two E6 strains formed, although no positive selection site was found. The 11 exclusive mutations on which selection force significantly changed were found in the 2C, 3AB and 3C genes.

**Conclusion:**

There are some E6 strains which did not cause the disease in the children of Yunnan. These E6 strains maybe come from a recombinant E6 strain which was associated with the outbreak of AFP in Shangdong in 2011. However, some new mutations were found in the 2C, 3AB and 3C genes of these asymptomatic strains, and these mutations may be constraint by the natural selection and could be potentially responsible for clinical presentations.

**Electronic supplementary material:**

The online version of this article (doi:10.1186/s12985-017-0809-2) contains supplementary material, which is available to authorized users.

## Background

Echovirus 6 (E6) belongs to the enterovirus B species. Enteroviruses (EV) are small non-enveloped viruses with a single-stranded positive-sense RNA. EV infections are usually asymptomatic, but some strains of EV are associated with outbreaks of serious diseases such as, acute flaccid paralysis (AFP), aseptic meningitis, encephalitis, and myocarditis [1]. Additionally, E6 infections are also associated with aseptic meningitis, herpangina, and epidemic pleurodynia based on the reports from enterovirus surveillance programs [2]. In 2011, E6 was also found in the oral epithelial cells of a hand, foot, and mouth disease (HFMD) patient [3] in China.

Similar to other EVs, such as EV71, the genome of E6 is approximately 7500 nucleotides and is comprised of a single open reading frame and 3′ and 5′ terminal untranslated regions (UTRs). There are 11 protein-coding genes in the ORF (open reading frame) [4]. Four genes (VP1, VP2, VP3 and VP4) encode the capsid proteins of the virus. The other genes encode viral proteins including two viral proteases (2A, and3C), an RNA-dependent-RNA-polymerase (3D), two proteins involved in RNA synthesis (2B, and 2C), and a primer for initiating RNA synthesis (3AB) [5]. Traditionally, EVs have been classified based on a neutralization test [6]. Currently, their classification is based on molecular typing of the VP1 gene. The strains with <70% VP1 nucleotide (nt) similarity are classified as different types, and strains with >75% nt similarity are classified as members of the same type [7, 8]. For strains with 70–75% nucleotide similarity, a more stringent value of 88% amino acid identity is more appropriate for their classification [9]. The genus *Enterovirus* was classified into 12 species, of which seven can infect humans (EV-A to D and rhinovirus-A to C) [10]. Enterovirus B (EV-B) is the most diverse species and is composed of at least 61 types, including E6 [11].

Echovirus 6 (E6) infections are associated with aseptic meningitis and acute flaccid paralysis (AFP). But most of infections are asymptomatic. The mechanism of E6 virulence is unknown. There is a wide variety of reasons that may affect whether or not the infected individual actually has any disease manifestations such as than virus genetics previous exposure to similar viruses, host genetics, age, nutrition etc. There is evidence that certain mutations are associated with changes in tissue tropism and virulence by comparing the genomes of Enteroviruses in mild and severe infections, such as EV71 [12]. But there is a little similar research on E6. Although it is known that E6 infections are usually asymptomatic, most studies have reported the gene sequences of pathogenic or disease-related strains. So in this study, some E6 strains from asymptomatic infections were isolated and sequenced to help us to find the mutations which could be potentially responsible for clinical presentations.

## Methods

### Sample collection and virus isolation

Stool samples of children were collected from three kindergartens in Kunming city, Yunnan province of China. The three kindergartens are located in the east, north and south of Kunming. The children were asymptomatic for virus infection and did not present with fever, cough, diarrhea or rash in the 2 weeks before and after sample collection. These children did not develop any diseases known to be caused by EV infection and did not have contact with patients recently infected by EVs. The children’s ages ranged from 3 to 6, and stool samples were collected between June and August in 2013.

The stool samples were collected and processed according to standard procedures recommended by the World Health Organization (WHO) [13]. RD (human rhabdomyosarcoma cells), KMB17 (human embryonic lung diploid fibroblast) and A549 (human lung cancer cells) cell lines were used to isolate the viruses [14]. Within three passages, samples that induced a cytopathic effect (CPE) were considered positive, and the supernatants were harvested and stored at −80 °C.

### Amplification and sequencing

The Viral RNA was extracted from cell culture supernatants using a QIAamp Viral RNA Mini Kit (QIAGEN, USA). RT-PCR was carried out using the PrimeScript One Step RT-PCR Kit Ver. 2 (TAKARA, China). Primer pairs 222 and 224 were used to amplify the partial VP1 gene [15], and primers for amplifying the complete genome were designed based on the published sequence of the D’Amori strain, and some primers were designed using the “primer-walking” strategy. The positive PCR products were sequenced at BGI Sequencing Company (Beijing, China). The whole genomes of these two strains were submitted to the Enterovirus Genotyping Tool v0.1 [16] for classification.

### Phylogenetic tree construction and recombination analyses

Viruses belonging to the same serotype as the newly identified E6 strains were chosen to construct the phylogenetic trees, and the sequences of these viruses were retrieved from GenBank. Their entire coding region of VP1 genes were used to construct the VP1 phylogenetic tree. For construction of the coding sequences (CDS) tree, the Enteroviruses that did not belong to the E6 serotype but whose gene was highly similar were also included. These non-E6 strains were selected by using each gene of K727 strain to compare with the sequences of EV database by BLAST. If the non-E6 strain contained a gene which has above 90% similarity with K727, this non-E6 strain was selected. The whole CDS sequences of these EV strains were aligned using Geneious 4.8 software [17]. Phylogenetic analyses were conducted using Molecular Evolutionary Genetic Analysis (MEGA) version 6 software [18]. The modified Nei-Gojobori method with a fixed Transition/Transversion ratio (0.5) was used to setup the model because the incidence of synonymous mutations and nonsynonymous mutations were significantly different in these viruses [19]. The neighbor-joining method was used to build the trees using 2000 bootstrap replicates. The nucleotide similarities among the strains were plotted with Simplot software version 3.5.1 [20], using a sliding window of 400 nucleotides moving in steps of 20 nucleotides by the Kimura 2-parameter method. The permuted tests were performed with the same software.

### Molecular evolution analyses

For discerning selection, a likelihood ratio test (LRT) [21] was used to compare the differences between non-synonymous and synonymous substitution rates. Non-synonymous mutations are much more likely to have an effect on fitness than synonymous changes. Thus, the ratio (*ω* = dN/dS) of the number of nonsynonymous substitutions per nonsynonymous site (dN) and the number of synonymous substitutions per synonymous site (dS) provides a sensitive measure of selective pressure at the protein level, with ω values <1, = 1, and >1 indicating purifying selection, neutral evolution, and positive selection, respectively [22]. This test was performed using CodeML of the PAML 4 software [23]. The phylogenetic trees required for the analysis were generated using MEGA6 via the neighbor-joining (NJ) method and the modified Nei-Gojobori method. The SITE, BRANCH and BRANCH-SITE models were all used in the analyses. The CodonFreq was set as F3X4, and the Small_Diff was set as 0.45e-6 in the CodeML model.

## Results

### Enterovirus cell culture and molecular classification

After culturing 96 stool specimens of healthy children with RD (human rhabdomyosarcoma cells), KMB17 (human embryonic lung diploid fibroblast) and A549 (human lung cancer cells) cell lines, two E6 strains were isolated. One was named K727/YN/CHN/2013 (abbreviated to K727), which was isolated from a 4.5-year-old boy using the KMB17 cell line. The other one was named K843/YN/CHN/2013 (K843), which was also isolated from a 4.3-year-old boy using the KMB17 cell line. The whole genomes of these two strains were sequenced. When the two sequences were submitted to the Enterovirus Genotyping Tool v0.1 [16], they were both classified into the E6 serotype with 100% bootstrap support. In addition, one Coxsackievirus A24 strain, 8 strains of Echovirus 12, one Echovirus 20 strain and one Enterovirus C99 strain were also isolated from these stool specimens.

### Phylogenetic tree construction

Three phylogenetic trees were constructed with the Neighbor-joining method using the modified Nei-Gojobori model [19]. One tree was built based on the VP1 sequences (Additional file 1: Figure S1). The VP1 sequences of 279 E6 strains were used to construct the tree, including K727, K843 and most E6 strains known in China. The K727 and K843 strains clustered together, and clustered with several KJ7724XX strains with 95% bootstrap support. These KJ7724XX strains were found in the AFP patients in Shangdong (a north province of China) in 2011, and the E6 strains were predominant in Shangdong in that year. In this branch, the other E6 strains in China all were found after 2011. Such as: KT633557 and KT633559 strains were found in Zhejiang province (central China) in 2013, LC120880 was found in Yunnan province (southwest China) in 2014 and KF487210 was found in Guangdong province (southern China) in 2012.

**Fig. 1:**
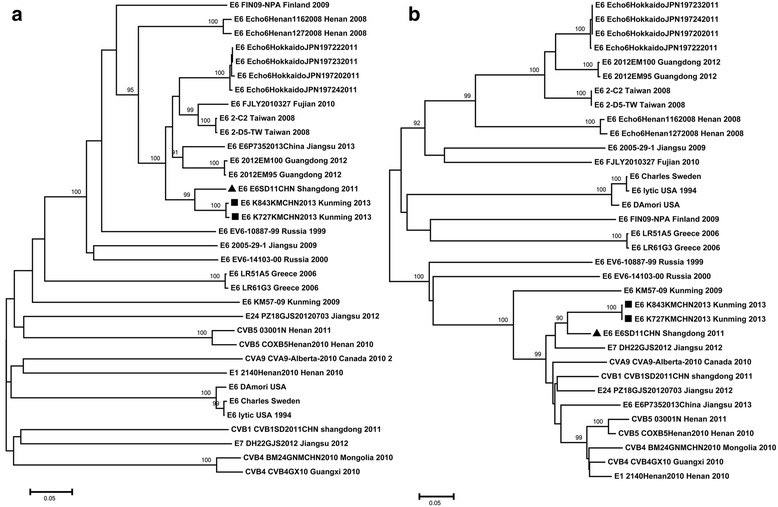
Polygenetic tree based on the CDS of EVs. **a** a tree was constructed based on the CDS of capsid coding region. **b** a tree was constructed based on the CDS of 3D gene. The two trees include all of the E6 strains with full CDS and some strains that have high similarity with K727 and K843. The *black circles* indicate the strains that we found, and the *black triangles* indicate the closest strains to K727 and K843

Another two trees were built using viral CDS of capsid coding region and 3D region separately (Fig. 1), because a potential recombination occurred in the 2C gene of K727 and K843. This tree included all E6 strains that have a complete CDS in GenBank as well as EV strains whose genes have high nt similarity with K727 and K843 (See Method). K727, K843 and E6SD11CHN clustered together as a novel branch with a bootstrap value of 99% for both trees. The capsid coding region of these three strains clustered with the E6 strains, while their 3D regions clustered with the non-E6 strain. So it looks like a recombination between EV types happened. Additionally, another E6 strain, E6/P735/2013/China, is showed the similar pattern.

### Similarity and recombination analyses

**Fig. 2:**
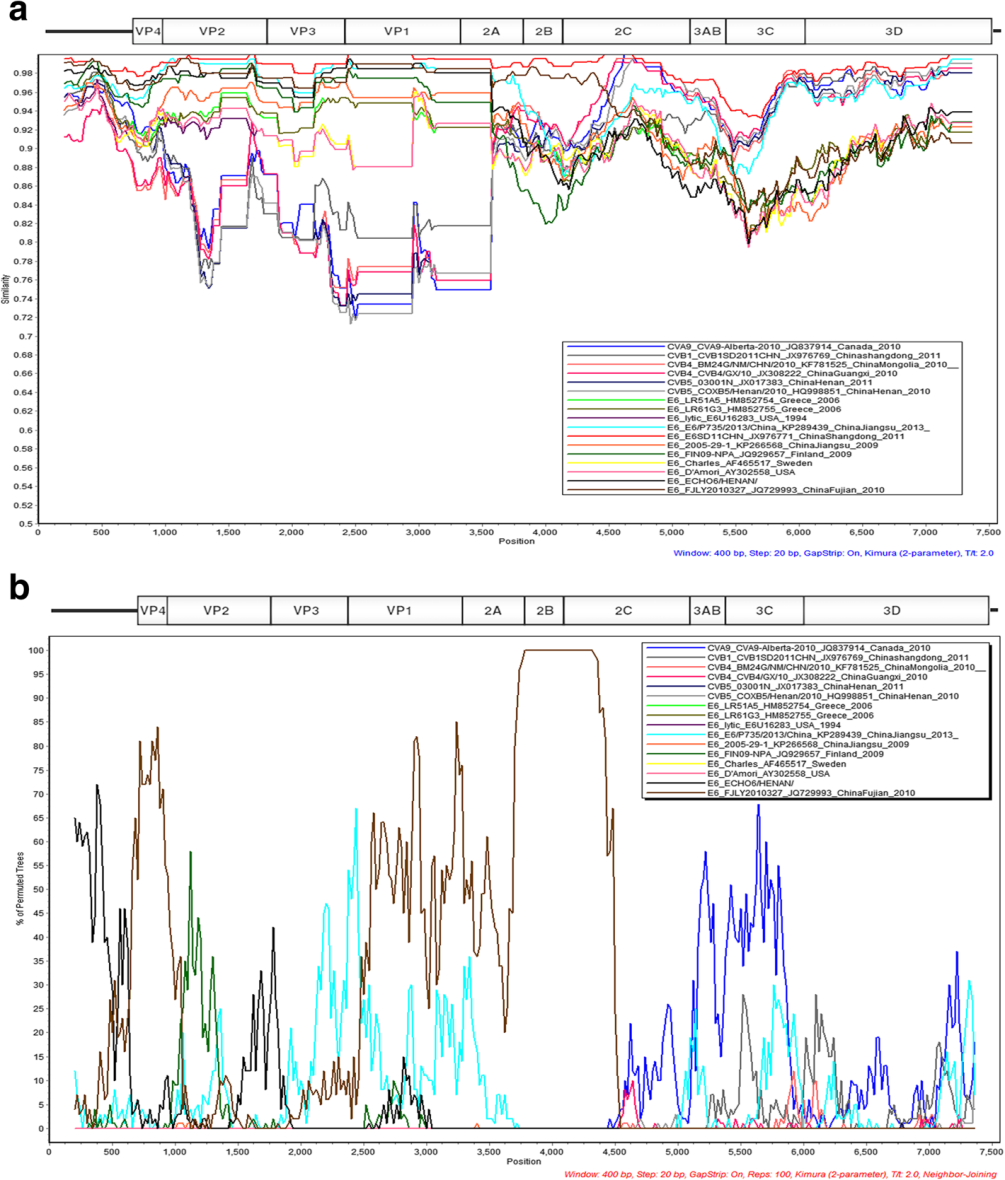
Similarity plot and bootscanning analysis queried by K727. **a** The similarity plot between K727 and other EVs. **b** The bootscanning analysis for K727. The analyses were conducted via Simplot v3.5.1 using a sliding window of 400 nucleotides, moving in steps of 20 nucleotides

A similarity comparison was plotted using simPlot 3.5.1 software [20], and the result from using K727 as a query is shown in Fig. 2a. Seventeen representative strains whose genes were most similar to K727 were selected in the comparison because if too many strains are used, the colors of some strains were too similar to be easily distinguished. In Fig. 2a, the region before the 2C gene of K727 is similar to that of the E6 serotype, and most E6 strains and K727 are more than 90% similar. In contrast, the region after the 2C gene of K727 has more than 90% similarity to non-E6 strains. The similarities of this region between most E6 strains and K727 are under 90%, which suggests a potential recombination in the 2C gene of K727.

Two E6 stains, E6SD11CHN and E6/P735/2013/China, which clustered with K727 and K843 in the both phylogenetic trees, were also highly similar to K727 on the whole genome level, especially E6SD11CHN (red line in Fig. 2a). Most of its genes had more than 95% similarity to K727 except for 3AB and 3C.

A bootscanning analysis was also performed on these strains. (E6SD11CHN was excluded because its high similarity could disrupt the analysis.) The result is shown in Fig. 2b. A significant change in the permutation percentage was observed in the middle of the 2C gene. Before this recombination site, strains K727 appears to share a common ancestor with another E6 strain (FJLY2010327). However, after this point, the K727 sequence becomes similar to a coxsackievirus A9 strain (CVA9-Alberta-2010), suggesting that a potential recombination occurred in the 2C gene of K727. The results of the similarity and recombination analyses for K843 were similar to those of K727 and can be found in Additional file 1: Figure S2.

### Molecular evolution analysis

Likelihood ratio tests (LRTs) were used to compare the different evolutionary scenarios with preset parameters to determine whether positive selection occurred. These were performed using PAML 4 software [23]. The strains which were used to construct the phylogenetic trees were used to perform the test. To begin with, two scenarios were envisioned. One (scenario 1) assumed that the positive selection occurred at some sites of the gene (positive selection model M2a), while the null model was M1a (nearly neutral), which assumed that there were no positive selection site in the gene, and only two kinds of sites in proportions *p*0 and *p*1 = 1 − *p*0 with 0 < *ω*0 < 1 (under purifying selection) and *ω*1 = 1 (neutral sites) [24]. The log-likelihood values, *p* and *ω* of these two models, were calculated using the SITE model of PAML 4, and the results are listed in Table 1. For every gene, the log-likelihood values of these two models were identical. Therefore, no positive selection site was found when the whole group was used. Actually, we found that most of the virus’ genes were under a strong purifying selection. The *ω*0 (Table 1) of these genes were only 0.006 to 0.022. Only 0.6 to 3.8% (*p*1) of the amino acids were nearly neutral (*ω*1 = 1), and they are also listed in Table 1.

**Table 1:** Log-likelihood values and parameter estimates for each gene of Echovirus E6 and some enterovirus B strains that could recombine with E6

Genes	Model code^a^	ℓ^b^	Parameter estimates^c^	Neutral sites^d^
VP4	**M1a**(nearly neutral)	−1073.6	*p*0 = 0.963, *p*1 = 0.037	18 N, 64S
			*ω*0 = 0.014, *ω*1 = 1.000	
	**M2a**(positive selection)	−1073.6	*p*0 = 0.963, *p*1 = 0.037, *p*2 = 0.000	
			*ω*0 = 0.014, *ω*1 = 1.000, *ω*2 = 4.752	
	**M0**(one *ω*)	−1076.3	*ω =* 0.021	
	**Branch(** *ω changed* **)**	−1076.2	*ω =* 0.021, *ω*(KM branch) *=* 0.0001	
VP3	**M1a**(nearly neutral)	−3343.7	*p*0 = 0.984, *p*1 = 0.016	35 N, 80A, 232R
			*ω*0 = 0.006, *ω*1 = 1.000	
	**M2a**(positive selection)	−3343.7	*p*0 = 0.984, *p*1 = 0.016, *p*2 = 0.000	
			*ω*0 = 0.006, *ω*1 = 1.000, *ω*2 = 5.550	
	**M0**(one *ω*)	−3353.0	*ω =* 0.010	
	**Branch(** *ω changed* **)**	−3351.5	*ω =* 0.009, *ω*(KM branch) *=* 0.048	
VP2	**M1a**(nearly neutral)	−3769.9	*p*0 = 0.977, *p*1 = 0.023	55Q, 137 V, 163 T, 165G
			*ω*0 = 0.010, *ω*1 = 1.000	
	**M2a**(positive selection)	−3769.9	*p*0 = 0.977, *p*1 = 0.023, *p*2 = 0.000	
			*ω*0 = 0.010, *ω*1 = 1.000, *ω*2 = 6.220	
	**M0**(one *ω*)	−3777.8	*ω =* 0.015	
	**Branch(** *ω changed* **)**	−3777.2	*ω =* 0.015, *ω*(KM branch) *=* 0.0001	
VP1	**M1a**(nearly neutral)	−4171.4	*p*0 = 0.991, *p*1 = 0.009	139A
			*ω*0 = 0.014, *ω*1 = 1.000	
	**M2a**(positive selection)	−4171.4	*p*0 = 0.991, *p*1 = 0.009, *p*2 = 0.000	
			*ω*0 = 0.014, *ω*1 = 1.000, *ω*2 = 33.127	
	**M0**(one *ω*)	−4177.0	*ω =* 0.016	
	**Branch(** *ω changed* **)**	−4177.0	*ω =* 0.016, *ω*(KM branch) *=* 0.023	
2A	**M1a**(nearly neutral)	−2500.2	*p*0 = 0.962, *p*1 = 0.038	7A, 14I, 23 N, 49 T, 63 V
			*ω*0 = 0.020, *ω*1 = 1.000	
	**M2a**(positive selection)	−2500.2	*p*0 = 0.962, *p*1 = 0.023, *p*2 = 0.015	
			*ω*0 = 0.014, *ω*1 = 1.000, *ω*2 = 1.000	
	**M0**(one *ω*)	−2533.4	*ω =* 0.032	
	**Branch(** *ω changed* **)**	−2532.3	*ω =* 0.031, *ω*(KM branch) *=* 0.090	
2B	**M1a**(nearly neutral)	−1743.4	*p*0 = 0.994, *p*1 = 0.006	
			*ω*0 = 0.022, *ω*1 = 1.000	
	**M2a**(positive selection)	−1743.4	*p*0 = 0.994, *p*1 = 0.006, *p*2 = 0.000	
			*ω*0 = 0.022, *ω*1 = 1.000, *ω*2 = 5.357	
	**M0**(one *ω*)	−1743.6	*ω =* 0.023	
	**Branch(** *ω changed* **)**	−1743.4	*ω =* 0.023, *ω*(KM branch) *=* 0.0001	
2C	**M1a**(nearly neutral)	−6875.4	*p*0 = 0.991, *p*1 = 0.009	34I, 48S
			*ω*0 = 0.008, *ω*1 = 1.000	
	**M2a**(positive selection)	−6875.4	*p*0 = 0.991, *p*1 = 0.009, *p*2 = 0.000	
			*ω*0 = 0.008, *ω*1 = 1.000, *ω*2 = 17.307	
	**M0**(one *ω*)	**−6889.1**	*ω =* 0.010	
	**Branch(** *ω changed* **)**	**−6882.4**	*ω =* 0.009, *ω*(KM branch) *=* 0.1052	
3AB	**M1a**(nearly neutral)	−2355.5	*p*0 = 0.977, *p*1 = 0.023	41K, 48I
			*ω*0 = 0.020, *ω*1 = 1.000	
	**M2a**(positive selection)	−2355.5	*p*0 = 0.977, *p*1 = 0.029, *p*2 = 0.000	
			*ω*0 = 0.020, *ω*1 = 1.000, *ω*2 = 12.815	
	**M0**(one *ω*)	**−2356.4**	*ω =* 0.024	
	**Branch(** *ω changed* **)**	**−2348.1**	*ω =* 0.021, *ω*(KM branch) *=* 0.445	
3C	**M1a**(nearly neutral)	−3926.0	*p*0 = 0.992, *p*1 = 0.008	57 V
			*ω*0 = 0.016, *ω*1 = 1.000	
	**M2a**(positive selection)	−3926.0	*p*0 = 0.992, *p*1 = 0.008, *p*2 = 0.000	
			*ω*0 = 0.016, *ω*1 = 1.000, *ω*2 = 2.687	
	**M0**(one *ω*)	**−3929.2**	*ω =* 0.018	
	**Branch(** *ω changed* **)**	**−3923.8**	*ω =* 0.016, *ω*(KM branch) *=* 0.1411	
3D	**M1a**(nearly neutral)	−8184.6	*p*0 = 0.989, *p*1 = 0.011	32H, 75R, 259S, 435S
			*ω*0 = 0.014, *ω*1 = 1.000	
	**M2a**(positive selection)	−8184.6	*p*0 = 0.989, *p*1 = 0.011, *p*2 = 0.000	
			*ω*0 = 0.014, *ω*1 = 1.000, *ω*2 = 33.13	
	**M0**(one *ω*)	−8217.2	*ω =* 0.017	
	**Branch(** *ω changed* **)**	−8217.1	*ω =* 0.017, *ω*(KM branch) *=* 0.025	

^a^Two types of hypotheses were tested for every gene. One is that positive selection happened at some sites (M2a) compared with its null model (M1a), which is that every site in the gene is nearly neutral. The likelihood values of this hypothesis were calculated using the site model in the PAML software. The other hypothesis is that the *ω* changed for the nonpathogenic branch (Branch Model) compared with its null model (M0), indicating the *ω* remained unchanged in the whole tree. The likelihood values of this hypothesis were calculated by using the branch model in the PAML software

^b^The bold ℓ means that there is a significantly different Log-likelihood value between the Branch and M0 models

^c^ *ω* is the ratio of nonsynonymous to synonymous substitutions (*d*_N_/*d*_S_) of sites. *ω*0, with the proportion of *p*0, comprises the sites at which nonsynonymous mutations are “slightly deleterious”; *ω*1 is the *d*_N_/*d*_S_ of completely neutral sites (*ω*1 = 1) with a proportion of *p*1; *ω*2 is the *d*_N_/*d*_S_ of positively selected sites with a proportion of *p*2

^d^The Naive Empirical Bayes (NEB) probabilities indicated that these sites are neutral with a possibility above 90%

Positive selection often acts on only a few sites and only for a short period of evolutionary time. Scenario 1 tested positive selection in all individuals of every branch, and the signal may be swamped by ubiquitous negative selection. Scenario 2 was considered because we wanted to determine whether adaptive evolution occurred in the asymptomatic E6 strains. The hypothesis in scenario 2 is that the *ω* changed when the asymptomatic branch (KM branch clustered with K737 and K843 in Fig. 1) grew, and this model was called the Branch (*ω* changed) model. Its null model was the M0 model (one *ω*), which assumed that *ω* was unchanged in the whole tree. The log-likelihood values, *p* and *ω* of these two models, were calculated using the BRANCH model [25] of PAML 4, and the results are listed in Table 1. The two models’ log-likelihood values for VP4, VP2, VP1, 2B and 3D were almost the same, so the *ω* for these genes in the KM branch were not significantly different. The log-likelihood values of VP3 and 2A have slight differences, but they are not significant. For example, the VP3 gene has an LRT statistic of 2Δℓ = 2 × ((−3351.5) - (−3353.0)) = 2 × 1.5 = 3, with a P of 0.08 and a df = 1 using the chi-square test [26]. On the contrary, the LRTs of 2C, 3AB and 3C genes had significantly different *ω* values on the KM branch. The LRT statistic of 2C was 2Δℓ = 2 × ((−6882.4) - (−6889.1)) = 2 × 6.7 = 13.4, and *P* = 2.5 × 10^−4^ with df = 1. The LRT statistic of 3AB was 2Δℓ = 2 × ((−2348.1) - (−2356.4)) = 2 × 8.3 = 16.6, and *P* = 4.6 × 10^−5^ with df = 1. The LRT statistic of 3C was 2Δℓ = 2 × ((−3923.8) - (−3929.2)) = 2 × 5.4 = 10.8, and *P* = 0.001 with df = 1. We also noticed that the *ω* of the KM branch was 8 to 20 times the *ω* of the whole tree, which may imply that evolution accelerated when the asymptomatic branch developed.

Since the *ω* increased on the KM branch for 2C, 3AB and 3C genes, a test was performed to determine whether there were sites under positive selection along this lineage in these three genes. Thus, scenario 3 was set up. The branches of the tree were divided a priori into foreground and background lineages, and their evolution parameters were set according to the branch-site model A, suggested by Yang [27]. The hypothesis (H1) in scenario 3 is that there were some sites under positive selection when the KM branch (foreground lineage) formed, and its null model (H0) assumed that positive selection did not happen along this lineage, in other words, there is only one *ω* for the whole tree. The simulations were conducted using the BRANCH-SITE model in PAML 4, and the results are listed in Table 2. Unfortunately the log-likelihood values between H0 and H1 were almost identical, and we did not find sites under positive selection along this lineage for these three genes. However, we did find that some sites that were under a strong purifying selection became neutral on this branch. The *ω* (*ω*2a in Table 2) of these sites increased from 0.01 or 0.02 (foreground) to 1 (background). The sites with altered *ω* values were tested using a Naive Empirical Bayes (NEB) method with probabilities above 90% and are listed in Table 2. Eleven were only found in the genes of the K727 and K843 strains and are shown in bold.

**Table 2:** Log-likelihood values and parameter estimates for genes which showed increased *ω* on the nonpathogenic KM branch

Genes	Model^a^	ℓ	Parameter estimates^b^	Sites with *ω2a* changed on the KM branch^c^
2C	**H0**	−6933.9	*p*0 = 0.88, *p*1 = 0.01, *p*2a = 0.11, *p*2b = 0.00	260 V, **307 L**, **312 T**, **319I**, **321R**
			Background*: ω*0 = 0.01, *ω*1 = 1.00, *ω2a* = 0.01, *ω2b* = 1.00	
			Foreground: *ω*0 = 0.01, *ω*1 = 1.00, *ω2a* = 1.00, *ω2b* = 1.00	
	**H1**	−6933.9	*p*0 = 0.88, *p*1 = 0.01, *p*2a = 0.11, *p*2b = 0.00	
			Background*: ω*0 = 0.01, *ω*1 = 1.00, *ω2a* = 0.01, *ω2b* = 1.00	
			Foreground: *ω*0 = 0.01, *ω*1 = 1.00, *ω2a* = 1.00, *ω2b* = 1.00	
3AB	**H0**	−2348.1	*p*0 = 0.52, *p*1 = 0.01, *p*2a = 0.47, *p*2b = 0.00	**15D,** 16 T, **29 L**, 41 K, **55D**
			Background*: ω*0 = 0.02, *ω*1 = 1.00, *ω2a* = 0.02, *ω2b* = 1.00	
			Foreground: *ω*0 = 0.02, *ω*1 = 1.00, *ω2a* = 1.00, *ω2b* = 1.00	
	**H1**	−2348.1	*p*0 = 0.52, *p*1 = 0.01, *p*2a = 0.47, *p*2b = 0.00	
			Background*: ω*0 = 0.02, *ω*1 = 1.00, *ω2a* = 0.02, *ω2b* = 1.00	
			Foreground: *ω*0 = 0.02, *ω*1 = 1.00, *ω2a* = 1.00, *ω2b* = 1.00	
3C	**H0**	−3919.5	*p*0 = 0.85, *p*1 = 0.01, *p*2a = 0.15, *p*2b = 0.00	**3P, 16G**, **52H**, **84 M**, 98S
			Background*: ω*0 = 0.01, *ω*1 = 1.00, *ω2a* = 0.01, *ω2b* = 1.00	
			Foreground: *ω*0 = 0.01, *ω*1 = 1.00, *ω2a* = 1.00, *ω2b* = 1.00	
	**H1**	−3919.2	*p*0 = 0.93, *p*1 = 0.01, *p*2a = 0.06, *p*2b = 0.00	
			Background*: ω*0 = 0.01, *ω*1 = 1.00, *ω2a* = 0.01, *ω2b* = 1.00	
			Foreground: *ω*0 = 0.01, *ω*1 = 1.00, *ω2a* = 3.31, *ω2b* = 3.31	

^a^H1 assumed there was positive selection at only some sites on the foreground branch (nonpathogenic branch), and H0 was its null model

^b^ *ω* is the ratio of nonsynonymous-synonymous substitutions (*d*_N_/*d*_S_) of sites, and *p* corresponded to their proportions. These parameters were set according to the branch-site model in the PAML software

^c^The mutations written in bold were only found in the KM branch, and these mutations were named in reference to K727. The amino acid positions are counted separately for each gene

## Discussion

E6 was the fifth most frequently detected EV in the United States during 1970–2005 and accounted for 6.2% of reported EV infections with a known serotype [1]. From 2006 to 2008, E6 was the second most frequently detected EV and accounted for 10.7% of reported EV infections [28]. In Spain, the prevalence of E6 was approximately 12.5%, but there was a substantial increase in the incidence of E6 detected (60%) in 2009 [29]. Therefore, E6 infections have been detected frequently during the past several decades and occasional spikes in the incidence of infection have occurred. In 2011, environmental surveillance data from Shangdong also indicated a significant increase in the detection rate of E6. There were 66 E6 strains in the 233 nonpolio enteroviruses (NPEVs) found in sewage samples (28%) [30]. In 2011, the incidence of E6 associated AFP was also very high (20%, 8/41) compared with other years or other EV types [31]. So we believed that the E6 outbreak happened or at least E6 predominated in Shangdong in 2011.

Based on the similarity and CDS polygenetic trees analyses, the E6 strain E6SD11CHN was the most similar to the K727 and K843 strains (about 95% similarity), and the two asymptomatic strains grouped with E6SD11CHN in the both trees of capsid coding region and 3D region. In the VP1-based polygenetic tree (Additional file 1: Figure S1), the K727 and K843 also grouped with E6SD11CHN and other strains with the accession number KJ7724XX with a 94% bootstrap value. These KJ7724XX strains are the exact E6 strains isolated from AFP patients in 2011 in Shangdong [31], and the E6SD11CHN was found in the oral epithelial cells of a HFMD patient in the Shangdong province of China in 2011 too [3]. So it suggested that the K727 and K843 should derive from the E6 strains of the 2011 Shangdong epidemic, such as E6SD11CHN. Additionally, a recombination happened when the E6SD11CHN formed, and it made the K727, K843 and E6SD11CHN grouped together and composed an independent novel branch. We also noticed that the E6/P735/2013/China was showed the similar pattern with these three strains. Interestingly, the E6/P735/2013/China was also isolated from a HFMD patient in Jiangsu province in 2013 [32]. To our knowledge, there is no report can confirm the E6 can cause the HFMD. So it is worth investigating the relationship between the strains in the novel branch and HFMD.

In this work, we attempted to identify mutated sites that mutations could be potentially responsible for clinical presentations by comparing mutations between symptomatic and asymptomatic viruses. A positive selection can be tested by comparing the ratio of non-synonymous and synonymous mutations. Therefore, LRTs were used to locate these mutations. When tests were performed on the whole tree, no positive selection site was found. However, a strong purifying selection acted on more than 96% of coding sites in all of the viral genes, and most of amino acid of these viruses tended to be conserved in evolution. This finding was similar to another study [33]. However, when we just focused on the asymptomatic KM branch, the *ω* of 2C, 3AB and 3C genes on the KM branch were significantly increased, indicating the evolution of these genes accelerated and more nonsynonymous mutations occurred on these genes. Unfortunately, BRANCH-SITE analyses failed to identify significant positive selection in these three genes. However, we did find some sites whose *ω* significantly changed in 2C, 3AB and 3C genes, and 11 of them were only found in the asymptomatic strains (K727 and K843). These mutations may be potentially responsible for clinical presentations.

2C, 3AB and 3C genes encode the non-structural proteins of EVs. They play very important roles in viral genome amplification and translation [34]. Protein 2C is required for intracellular membrane rearrangement and for the formation of virus-induced cytoplasmic vesicles [35] and RNA replication [36], and it has ATPase activity [37]. The four exclusive mutations (307 L, 312 T, 319I and 321R) whose *ω* changed on the KM branch were found in the 2C gene. They are located on a putative amphipathic helices site at the C-terminus of the 2C protein, but this region does not seem as important as the other N-terminal putative amphipathic helices that play an important role in membrane binding [38].

The 3A protein is known to inhibit both major histone class I expression and intracellular membrane transport, which may be important for evasion of the immune response by picornaviruses [34, 39]. Three exclusive mutations (15D, 29 L, and 55D) were found in 3AB, and they were all located on the P3A domain (amino acid 1 to 59) of the 3A protein. Because the 3A protein plays an important role in evasion of the immune response, we think that these mutations might be the potential “virulence associated mutations” for which we were searching. In EV71, Wen, H. L. et al. also suggested several mutations of 3A might be potential virulent positions too [40].

The 3C proteins are multifunctional and are involved in viral protein processing, host protein cleavage and RNA replication [34]. Four exclusive mutations (13P, 16G, 52H and 84 M) were found in the 3C protein, and they were also located on its Peptidase_C3 domain. In the EV71, some reports also showed the mutations in the 3C gene are potentially associated with the neurovirulence of EV71 [41] or might be potential virulent positions [40]. So the functions of these mutations are still unknown and deserve further investigation in vitro and in vivo, particularly for the 3A and 3C gene.

## Conclusion

We found two asymptomatic E6 strains in the healthy children in the Yunnan. These strains may be derived from KJ7724XX strains, which were predominant in AFP patients in Shangdong in 2011. Molecular evolution analyses showed that the evolution was accelerated when these two E6 strains formed and the selection force may act on the 2C, 3AB and 3C genes of these strains. Some new mutations were found in the 2C, 3AB and 3C genes of these asymptomatic strains, and these mutations may be constraint by the natural selection and could be potentially responsible for clinical presentations.

## Additional file

Additional file 1: Figure S1. Polygenetic tree based on the VP1 sequences of EVs. The tree includes nearly all of the E6 strains found in China by 2016. The black circles indicate the strains that we found, and the black triangles indicate the closest strains to them. **Figure S2.** Similarity plot and bootscanning analysis queried by K843. (a) The similarity plot between K843 and other EVs. (b) The bootscanning analysis for K843. The analyses were conducted via Simplot v3.5.1 using a sliding window of 400 nucleotides moving in steps of 20 nucleotides. (PDF 919 kb)

## Abbreviations

AFP: Aseptic meningitis and acute flaccid paralysis
CPE: Cytopathic effect
E6: Echovirus 6
EV: Enteroviruses
EV-B: Enterovirus B
K727: K727/YN/CHN/2013
K843: K843/YN/CHN/2013
LRT: Likelihood ratio test
NJ: Neighbor-joining
nt: Nucleotide
ORF: Open reading frame
RT-PCR: Reverse-transcription polymerase chain reaction
UTR: Untranslated regions
WHO: World Health Organization
