# Supplementary material for: Molecular evolution of two asymptomatic echovirus 6 strains that constitute a novel branch of recently epidemic echovirus 6 in China
Source: Virol J. 2017 Jul 25;14:140. doi: 10.1186/s12985-017-0809-2 (PMC5526271; doi:10.1186/s12985-017-0809-2)
Supplement: Additional file 1: Figure S1. — Polygenetic tree based on the VP1 sequences of EVs. The tree includes nearly all of the E6 strains found in China by 2016. The black circles indicate the strains that we found, and the black triangles indicate the closest strains to them. Figure S2. Similarity plot and bootscanning analysis queried by K843. (a) The similarity plot between K843 and other EVs. (b) The bootscanning analysis for K843. The analyses were conducted via Simplot v3.5.1 using a sliding window of 400 nucleotides moving in steps of 20 nucleotides. (PDF 919 kb) [file 12985_2017_809_MOESM1_ESM.pdf]

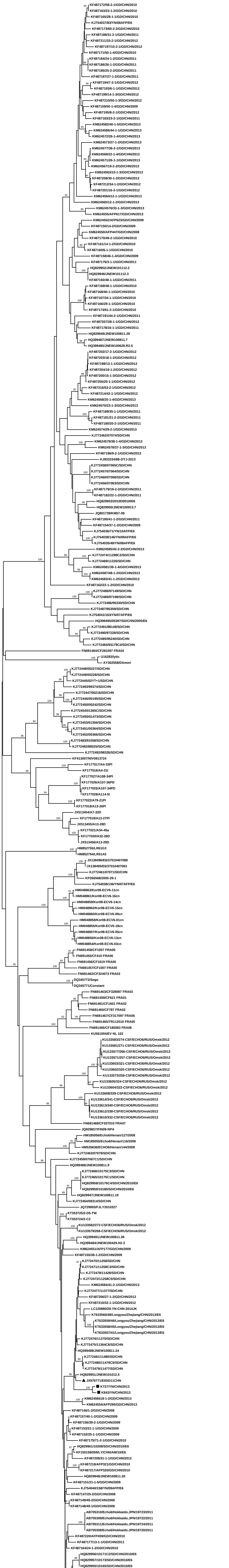

Supplementary Figure 1. Polygenetic tree based on the VP1 sequences of EVs. The tree includes nearly all of the E6 strains found in China by 2016. The black circles indicate the strains that we found, and the black triangles indicate the closest strains to them.

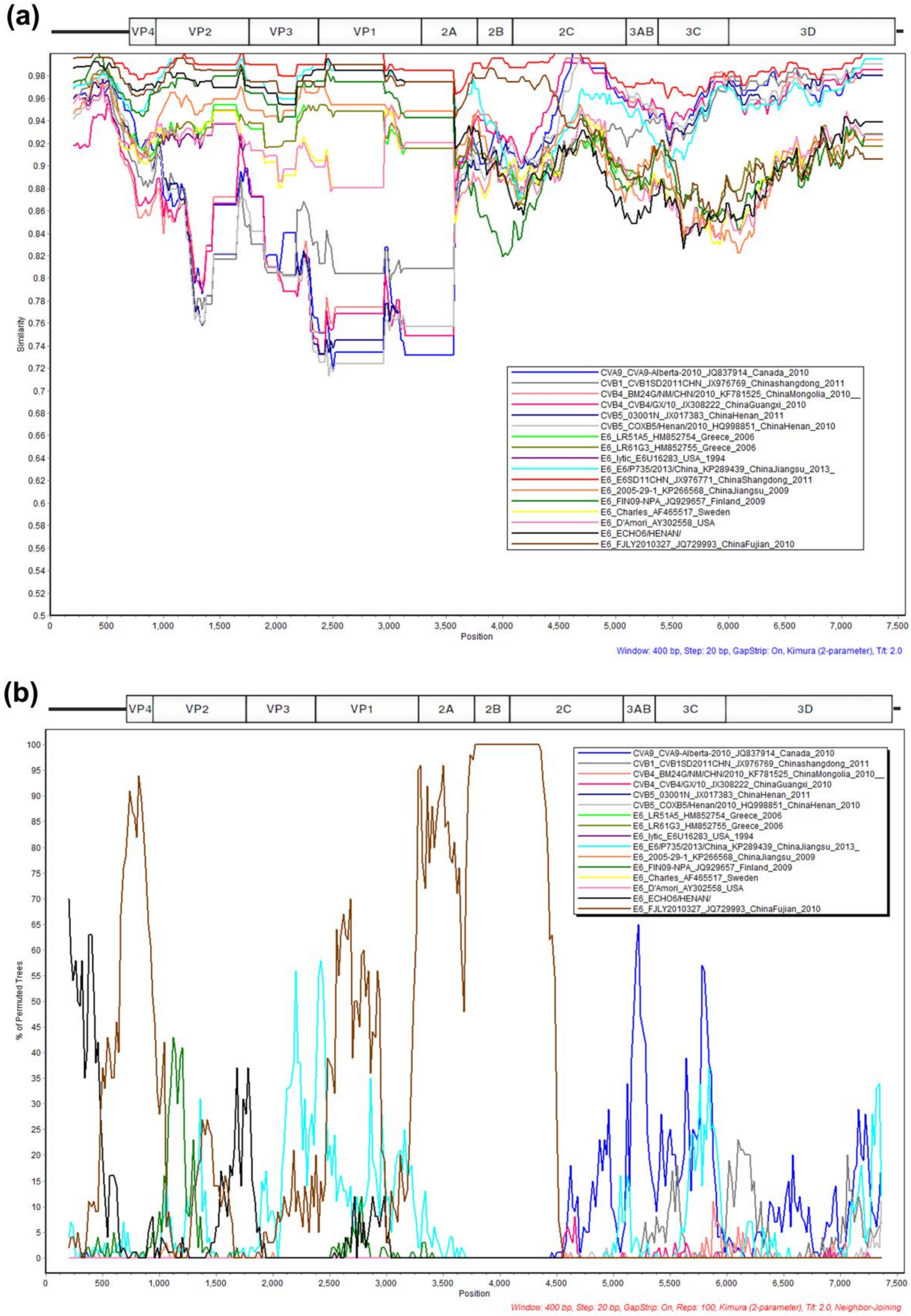

Supplementary Figure 2. Similarity plot and bootscanning analysis queried by K843. (a) The similarity plot between K843 and other EVs. (b) The bootscanning analysis for K843. The analyses were conducted via Simplot v3.5.1 using a sliding window of 400 nucleotides moving in steps of 20 nucleotides.
